# How do impairments in cognitive functions affect activities of daily living functions in older adults?

**DOI:** 10.1371/journal.pone.0218112

**Published:** 2019-06-07

**Authors:** Meng-Ta Lee, Yuh Jang, Wan-Ying Chang

**Affiliations:** 1 School of Occupational Therapy, College of Medicine, National Taiwan University, Taipei, Taiwan; 2 Division of Occupational Therapy, Taipei Hospital, Ministry of Health and Welfare, New Taipei City, Taiwan; 3 Division of Occupational Therapy, Department of Physical Medicine and Rehabilitation, National Taiwan University Hospital, Taipei, Taiwan; Universita degli Studi Europea di Roma, ITALY

## Abstract

The assessment of daily living activities could provide information about daily functions and participation restrictions to develop intervention strategies. The purposes of this study were to assess the scores of the Barthel Index (BI) and Lawton Instrumental Activities of Daily Living (IADL) scale in older adults with cognitive impairment and to explore the different effects that levels of cognitive functions have on changes in IADL functions. We recruited 31 participants with dementia, 36 with mild cognitive impairment (MCI), and 35 normal controls (NCs) from the neurology outpatient department of a regional hospital. The results of the demographic and clinical characteristics through the Lawton IADL scale, BI, Quick Mild Cognitive Impairment (Q*mci*) screen, Montreal Cognitive Assessment (MoCA), and Mini-Mental State Examination (MMSE), were collected on the same day and compared with the Kruskal–Wallis test, Wilcoxon rank-sum test, Fisher’s exact test, and a multiple linear regression analysis, as appropriate. In the BI, bathing was the most discriminating activity to differentiate patients with MCI and dementia; in the Lawton IADL scale, medication responsibility and shopping were the most discriminating activities to differentiate NCs and patients with MCI, and patients with MCI and dementia, respectively. In addition, the predictors of changes in Lawton IADL scale scores were the problem-solving score of the Clinical Dementia Rating scale, a Q*mci* score of > 20.4 and an age of ≤ 81.2 years, a MoCA score of < 9.4 and an age of > 81.2 years, and the MMSE score and an age of > 81.2 years. This study adds to the evidence that the description of basic and instrumental daily activities is integrated in older adults with cognitive impairment. Notably, the Q*mci* is the most significant predictor of changes in IADL function for “young” older adults, as are the MoCA and MMSE for “old” older adults.

## Introduction

The number of people older than 65 years has been increasing worldwide [1]. The global prevalence of dementia is 1.2%–7.2% [2], and the population of older adults with dementia is expected to increase concurrently with global aging. The reported proportion of individuals with mild cognitive impairment (MCI)—at 5.0%–36.7% [3]—is higher than that of people with dementia; however, most cases of MCI and dementia in older adults remain underdiagnosed and unidentified.

People with MCI are completely capable of conducting self-care activities; they exhibit a slight impairment in the instrumental activities of daily living (IADL), whereas people with dementia need assistance or at least supervision for the basic activities of daily living (BADL) [4–6]. Although cognitive tests are routinely administered and are useful for detecting MCI and dementia under clinical settings, activities reflecting daily living functions are more easily observed by carers in real-life circumstances. Moreover, the IADL and BADL scales are simpler and less confrontational in their evaluation; they not only provide information on daily living functions but also address the participation restrictions for further intervention.

The Lawton IADL scale [7] and the Barthel Index (BI) [8] are two of the most popular IADL and BADL scales, respectively. Previous studies have supported the use of the IADL scale to study older adults with dementia [9–11]. Daily living function activities are correlated with various cognitive instruments in older adults with cognitive impairment [12]. Furthermore, cognitive decline affects the performance of daily living activities in patients with MCI and dementia [4, 13]. The more cognitive functions decline, the more daily functions are inhibited. However, the association between cognitive functions and changes in the daily functions of older adults remains unknown. Predicting the effect of cognitive functions in changes of daily functions is essential not only for clinical practitioners to provide appropriate interventions to improve or maintain daily performances but also for carers to prepare sufficient socioeconomic resources to treat patients with MCI and dementia.

Therefore, the impact of different levels of cognitive functions on the changes in daily living activities in older adults with cognitive impairment must be explored in clinical practice. The research questions are as follows: (1) how different are the BI and Lawton IADL scale scores between normal controls (NCs), older adults with MCI, and older adults with dementia? and (2) how do different levels of cognitive function affect changes in the IADL functions of older adults with cognitive impairment? We hypothesize that (1) the BI and Lawton IADL scale scores are different between NCs, older adults with MCI, and older adults with dementia; that (2) different levels of cognitive function elicit different changes in the IADL functions. This study’s objectives were to assess the BI and Lawton IADL scale scores between NCs, older adults with MCI, and older adults with dementia and to explore the effect of different levels of cognitive function on changes in IADL functions.

## Materials and methods

### Participants

Similar to our previous study [12], participants aged ≥ 65 years either with MCI and dementia or without (NCs), who were able to follow instructions and understand the content of the assessment through verbal communication were recruited consecutively between May 2017 and December 2017 from the neurology outpatient department of a regional hospital in New Taipei City, Taiwan. The NCs, determined by the absence of subjective and objective cognitive complaints, were recruited using the convenience sampling method. According to the Diagnostic and Statistical Manual of Mental Disorders, Fourth Edition [14], National Institute of Neurological and Communicative Diseases and Stroke, Alzheimer's Disease and Related Disorders Association [15] criteria, and the National Institute on Aging and the Alzheimer’s Association workgroup’s diagnostic guidelines for Alzheimer’s disease [13], participants were diagnosed as having amnestic-type MCI and dementia (Alzheimer’s disease or vascular or mixed dementia subtypes) and were further classified using the Clinical Dementia Rating (CDR) scale global scores of 0.5 and 1–3, respectively [4]. Participants who had depression (according to the Geriatric Depression Scale Short Form score of > 7 points) [16] or were diagnosed with other subtypes of MCI or dementia (frontotemporal dementia or Parkinson’s disease or Lewy Body dementia) were excluded.

The study was approved by the Institutional Review Board of the Taipei Hospital, Ministry of Health and Welfare (TH-IRB-0016-0033), and written informed consents were obtained from the NCs themselves, and participants with MCI or dementia along with their legal guardians.

### Data collection

As in our previous cross-sectional study [12], we collected the demographic data, including age in years, gender, and years of education, and applied the Lawton IADL scale, BI, Quick Mild Cognitive Impairment screen (Q*mci*) screen [12], Montreal Cognitive Assessment (MoCA) [17], and Mini-Mental State Examination (MMSE) [18]. On the same day, the trained rater (Lee), blinded to final diagnosis, alternately and randomly administered the Q*mci*, MoCA, and MMSE to all participants themselves, and the Lawton IADL scale, and BI to the NCs themselves and participants with MCI or dementia along with their legal guardians. At the same time as the current score of the Lawton IADL scale was assessed, the Lawton IADL scale at baseline was also assessed to further calculate change in the scores of the Lawton IADL scale. The time of the baseline Lawton IADL scale referred to the time before the decline of IADL functions began in their own judgements.

### Instruments

The Lawton IADL scale is used to measure the instrumental activities of daily living independence, including telephone use, shopping, food preparation, medication responsibility, and finance-handling ability. The Lawton IADL scale scores range from 0 to 8; a higher score indicates greater independence in the complex activities of daily living [7].

The BI is used to measure activities reflecting the daily living independence, such as bathing, grooming, dressing, controlling bowels and bladder, using the toilet, and climbing stairs. The BI scores range from 0 to 20; a higher score indicates greater independence [8].

The Q*mci* is a performance test, standardized and validated to measure cognitive functions in orientation, memory, executive function, and visuospatial function. The Q*mci* scores range from 0 to 100; a higher score indicates greater cognitive function [12].

The MoCA is also a performance test, standardized and validated to measure cognitive functions in orientation, attention, language, memory, executive function, and visuospatial function. One point is added for individuals whose educational level is ≤ 12 in MoCA scoring. The MoCA scores range from 0 to 30; a higher score indicates greater cognitive function [17].

The MMSE is a performance test, standardized and validated to measure cognitive functions in orientation, attention, language, memory, and visuospatial function. The MMSE scores range from 0 to 30; a higher score indicates greater cognitive function [18].

### Statistical analysis

The IBM SPSS 19.0 software (IBM Corporation, Somers, NY, U.S.A.) and the R 3.4.3 software (R Foundation for Statistical Computing, Vienna, Austria) were used in statistical analysis. A two-sided *p* value of ≤ 0.05 was considered statistically significant. Continuous variables and categorical variables were presented as mean ± standard deviation (SD) and frequencies and percentages (%) respectively.

In univariate analysis, the unadjusted effect of each item of the BI and Lawton IADL scale was examined among the subjects of NC, MCI, and dementia using the Kruskal-Wallis test with the Dunn’s post hoc test for multiple comparisons. Then, the demographic and clinical characteristics were compared between the subjects of two age groups using Wilcoxon rank-sum test and chi-square test as appropriate for the data type. Next, multivariate analysis was conducted by fitting multiple linear regression model to estimate the adjusted effects of predictors on the change score of the Lawton IADL scale, where the change score of the Lawton IADL scale = the current score of the Lawton IADL scale − the Lawton IADL scale at baseline.

The goal of regression analysis was to find one or a few parsimonious regression models that fitted the observed data well for effect estimation and/or outcome prediction. To ensure a good quality of analysis, the model-fitting techniques for (1) variable selection, (2) goodness-of-fit (GOF) assessment, and (3) regression diagnostics and remedies were used in our regression analyses. Specifically, the stepwise variable selection procedure (with iterations between the forward and backward steps) was applied to obtain the best candidate final linear regression model using the My.stepwise package of R [19]. All the univariate significant and non-significant relevant covariates (listed in Table 1) and some of their interaction terms (i.e., moderators) were put on the variable list to be selected. The significance levels for entry (SLE) and for stay (SLS) were set to 0.15 for being conservative. Then, with the aid of substantive knowledge, the best candidate final linear regression model was identified manually by dropping the covariates with *p* value > 0.05 one at a time until all regression coefficients were significantly different from 0. Any discrepancy between the results of univariate analysis and multivariate analysis was likely due to the confounding effects of uncontrolled covariates in univariate analysis or the masking effects of intermediate variables (i.e., mediators) in multivariate analysis.

**Table 1:** Univariate analysis of the demographic and clinical characteristics between the subjects of two age groups.

Variable	Age ≤ 81.2 Years^a^ (*n* = 71)	Age > 81.2 Years^a^ (*n* = 31)	*p* Value
Age (years)	73.17 ± 4.67	86.29 ± 3.58	< 0.0001
Male (%)	32 (45.07%)	14 (45.16%)	0.9932
Years in education	7.68 ± 4.82	6.27 ± 4.91	0.1409
Q*mci*	48.75 ± 20.35	29.32 ± 19.04	< 0.0001
MoCA	18.89 ± 7.25	12.03 ± 7.02	< 0.0001
MMSE	24.31 ± 5.62	19.13 ± 6.55	< 0.0001
Barthel Index (BI)	7.68 ± 4.82	6.27 ± 4.91	< 0.0001
Lawton IADL scale	6.59 ± 2.03	4.06 ± 2.31	< 0.0001
Change score of the Lawton IADL scale^b^	-0.90 ± 1.70	-3.29 ± 2.40	< 0.0001

The listed sample statistics were mean ± standard deviation (SD) or frequency (percentage, %) as appropriate for the data type. The *p* values of the statistical tests were calculated using the Wilcoxon rank-sum test for continuous variables and the Fisher’s exact test for categorical variables.

**Abbreviations:** Q*mci* = Quick Mild Cognitive Impairment screen; MoCA = Montreal Cognitive Assessment; MMSE = Mini-Mental State Examination; and Lawton IADL scale = Lawton Instrument Activities of Daily Living scale.

**Fig 1:**
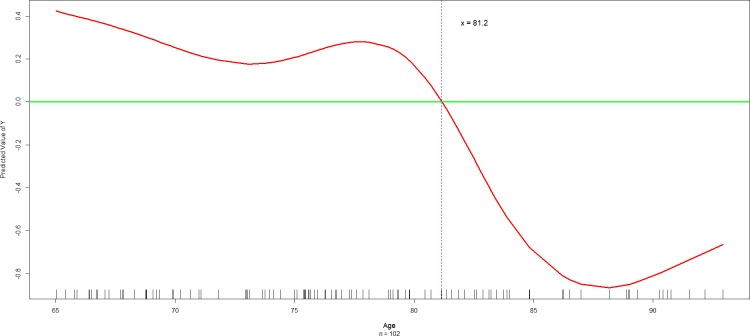
The plot of the fitted multiple generalized additive model (GAM) for estimating the partial effect of “Age” (years) on the “change score of the Lawton IADL scale”, adjusted for the effects of the “Problem solving score of the CDR” and the smoothed “MMSE score”. The *change score* of the Lawton IADL scale = The current score of the Lawton IADL scale − The Lawton IADL scale at baseline. Lawton IADL scale = Lawton Instrument Activities of Daily Living scale; CDR = Clinical Dementia Rating scale; and MMSE = Mini-Mental State Examination. The cut-off value of 81.2 years for “Age” was estimated by the intersection of the smoothed red curve and the green straight line at Predicted value of Y = 0. The many little vertical bars (called the *rugs*) right above the *X*-axis indicated where the observed values of “Age” located. The technical details of the GAM plot were described in the Materials and methods section.

^a^ As described in the Materials and methods section and shown in Fig 1, the cut-off value of 81.2 years for “age” was estimated by fitting generalized additive models (GAMs) during the stepwise variable selection procedure of regression analysis.

^b^ The *change score* of the Lawton IADL scale = The current score of the Lawton IADL scale − The Lawton IADL scale at baseline.

The coefficient of determination *R*^2^ was examined to assess the GOF of the fitted multiple linear regression model. Technically, the *R*^2^ statistic (0 ≤ *R*^2^ ≤ 1) equals the square of the Pearson correlation between the observed and predicted response values and it indicates how much of the response variability is explained by the covariates included in the multiple linear regression model. Simple and multiple generalized additive models (GAMs) were fitted to detect nonlinear effects of continuous covariates and identify appropriate cut-off point(s) for discretizing continuous covariates, if necessary, during the stepwise variable selection procedure. Computationally, the vgam() function (with the default values of smoothing parameters) of the VGAM package [20–22] was used to fit GAMs for our continuous outcome in R. Finally, the statistical tools of regression diagnostics for residual analysis, detection of influential cases, and check of multicollinearity were applied to discover any model or data problems. The values of variance inflating factor (VIF) ≥ 10 in continuous covariates or VIF ≥ 2.5 in categorical covariates indicate the occurrence of the multicollinearity problem among some of the covariates in the fitted regression model.

## Results

### Demographic and clinical characteristics

As demonstrated in our previous study [12], 119 participants were recruited, but 17 participants with depression were excluded. Within the remaining 102 participants (47 men and 55 women), 35 (34.3%) were NCs, 36 (35.3%) were diagnosed as having MCI, and 31 (30.4%) were diagnosed as having dementia. Participants with dementia (aged 82.11 ± 6.13 years) were significantly older than NCs (aged 73.64 ± 6.39 years; *p* < 0.001) or than those with MCI (aged 76.22 ± 7.41 years; *p* = 0.005). Participants with MCI (years of education: 6.83 ± 4.87; *p* = 0.007) and dementia (4.61 ± 4.35 years; *p* < 0.001) received significantly less education than NCs (10.03 ± 3.85 years). The mean Geriatric Depression Scale Short Form score among participants with dementia (3.84 ± 1.97 points) was significantly higher than that among NCs (1.83 ± 1.72 points; *p* < 0.001). Moreover, the Q*mci*, MoCA, and MMSE total scores of participants with dementia (18.44 ± 11.48; 8.13 ± 4.65; 15.26 ± 4.58, respectively) were significantly lower than those of NCs (64.06 ± 8.43; 24.51 ± 2.47; 28.29 ± 1.18, respectively) and patients with MCI (43.13 ± 14.71; 16.61 ± 5.05; 23.61 ± 4.02, respectively); however, the Q*mci*, MoCA, and MMSE total scores of participants with MCI were significantly lower than those of NCs (all *p*’s < 0.001).

Next, since age is a well-known predictor of both cognitive functions and the basic and instrumental activities of daily living functions, we compared the demographic and these clinical characteristics between two age groups in Table 1. As illustrated in Fig 1, the cut-off value, 81.2 years, for dichotomizing the continuous variable “Age” was estimated by fitting GAMs using a spline smoothing technique [17−19]. The total scores of the Q*mci*, MoCA, MMSE, BI, and Lawton IADL scale as well as the change score of Lawton IADL scale were all significantly higher in the subjects with age ≤ 81.2 years than those with age > 81.2 years.

### Regression analysis

As listed in Table 2, given the available demographic and clinical characteristics, including age, gender, years in education, total scores of Q*mci*, MoCA, MMSE, Lawton IADL scale at baseline, and the interaction terms between some of them, we identified four statistically significant predictors of the mean *change score* of the Lawton IADL scale in our stepwise linear regression analysis. Notice that the estimated intercept of the final multiple linear regression model was −2.4539, which means that while the values of all the other four covariates were zero, the current score of the Lawton IADL scale would on average reduce −2.4539 in our participants (Table 2). Then, after adjusting for the effects of the other covariates, an increment of one unit in the problem solving score of the CDR would on average reduce (−2.4539) + (−1.2425) × 1 in the current score of the Lawton IADL scale in our participants (Table 2). Similarly, the subjects with Age > 81.2 years and MoCA score < 9.4 would on average reduce (−2.4539) + (−1.2054) in the current score of the Lawton IADL scale (Table 2). However, the subjects with Age ≤ 81.2 years and Q*mci* score > 20.4 would on average reduce only (−2.4539) + 2.3674 in the current score of the Lawton IADL scale (Table 2). Finally, an increment of one unit in the MMSE score in subjects with Age > 81.2 years would on average reduce (−2.4539) + 0.0521 × 1 in the current score of the Lawton IADL scale (Table 2). The *R*^2^ of the final multiple linear regression model was 0.8230, which indicates a very good fit since the Pearson correlation between the observed and model-predicted change scores of the Lawton IADL scale was as high as 0.8230^½^ = 0.9072.

**Table 2:** Multivariate analysis of the predictors for the change score^a^ of the Lawton IADL scale by fitting multiple linear regression model with the stepwise variable selection procedure.

Covariate	Parameter Estimate	Standard Error	*t* Value	Pr > |*t*|
Intercept	-2.4539	0.4169	-5.8861	< 0.0001
Problem solving score of the CDR	-1.2425	0.1650	-7.5308	< 0.0001
Age ≤ 81.2 years^b^ × Q*mci* score > 20.4^b^^,^^c^	2.3674	0.4092	5.7857	< 0.0001
Age > 81.2 years^b^ × MoCA score < 9.4^b^^,^^c^	-1.2054	0.3791	-3.1795	0.0020
Age > 81.2 years^b^ × MMSE score^b^	0.0521	0.0177	2.9414	0.0041

**Abbreviations:** CDR = Clinical Dementia Rating scale; Q*mci* = Quick Mild Cognitive Impairment screen; MoCA = Montreal Cognitive Assessment; MMSE = Mini-Mental State Examination; and Lawton IADL scale = Lawton Instrument Activities of Daily Living scale.

^a^ The *change score* of the Lawton IADL scale = The current score of the Lawton IADL scale − The Lawton IADL scale at baseline.

^b^ The “Age ≤ 81.2 years”, “Age > 81.2 years”, “Q*mci* score > 20.4”, and “MoCA score < 9.4” are dummy variables with its value = 1 while the specified condition is satisfied and 0 otherwise. As described in the Materials and methods section, the cut-off value of 81.2 years for “age” was determined by fitting generalized additive models (GAMs) during the stepwise variable selection procedure of regression analysis.

^c^ The multiplication sign × is used to indicate an *interaction* between two covariates A and B and it means “A times B” mathematically. Thus, when both covariates are dummy variables, it can be directly interpreted as “and”―for example, “Age ≤ 81.2 years × Q*mci* score > 20.4” means the “Age ≤ 81.2 years and Q*mci* score > 20.4”.

**Goodness-of-fit assessment:** Given the sample size of 102, the *R*^2^ = 0.8230 indicates a very good fit since the Pearson correlation between the observed change score of the Lawton IADL scale and the model-predicted change score of the Lawton IADL scale was as high as 0.8230^½^ = 0.9072.

### Basic and instrumental activities of daily living functions

Following our previous study [12], we examined the differences in each item of the BI and Lawton IADL scale among the subjects of NC, MCI, and dementia from the same 102 participants in Tables 3 and 4. The total and most subtest scores of BI were significantly higher in the NCs and the subjects with MCI than those with dementia, except for the feeding and bowels. Similarly, the total and all subtest scores of the Lawton IADL scale were also significantly higher in the NCs and the subjects with MCI than those with dementia. By contrast, the food preparation and medication responsibility subtests of the Lawton IADL scale were significantly higher in the NCs than the subjects with MCI.

**Table 3:** Univariate analysis of the basic and instrumental activities of daily living functions among the subjects of NC, MCI, and dementia.

Characteristics	Total (*n* = 102)	NC (*n* = 35)	MCI (*n* = 36)	Dementia (*n* = 31)	*χ*^*2*^ (*df* = 2)	Pairwise Comparison^a^^,^^b^
**Barthel Index (BI)**	19.25 ± 2.32	19.89 ± 0.47	19.94 ± 0.33	17.74 ± 3.79	27.32**	1 > 3, 2 > 3
Feeding	1.98 ± 0.14	2.00 ± 0.00	2.00 ± 0.00	1.94 ± 0.25	4.63	
0	0 (0%)	0 (0%)	0 (0%)	0 (0%)		
1	2 (2%)	0 (0%)	0 (0%)	2 (6.5%)		
2	100 (98%)	35 (100%)	36 (100%)	29 (93.5%)		
Grooming	0.94 ± 0.24	1.00 ± 0.00	1.00 ± 0.00	0.81 ± 0.40	14.46*	1 > 3, 2 > 3
0	6 (5.9%)	0 (0%)	0 (0%)	6 (19.4%)		
1	96 (94.1%)	35 (100%)	36 (100%)	25 (80.6%)		
Toilet use	1.94 ± 0.28	2.00 ± 0.00	2.00 ± 0.00	1.81 ± 0.48	11.92*	1 > 3, 2 > 3
0	1 (1%)	0 (0%)	0 (0%)	1 (3.2%)		
1	4 (3.9%)	0 (0%)	0 (0%)	4 (12.9%)		
2	97 (95.1%)	35 (100%)	36 (100%)	26 (83.9%)		
Bathing	0.88 ± 0.32	0.97 ± 0.17	1.00 ± 0.00	0.65 ± 0.49	24.04**	1 > 3, 2 > 3
0	12 (11.8%)	1 (2.9%)	0 (0%)	11 (35.5%)		
1	90 (88.2%)	34 (97.1%)	36 (100%)	20 (64.5%)		
Dressing	1.91 ± 0.35	2.00 ± 0.00	2.00 ± 0.00	1.71 ± 0.59	17.03**	1 > 3, 2 > 3
0	2 (2%)	0 (0%)	0 (0%)	2 (6.5%)		
1	5 (4.9%)	0 (0%)	0 (0%)	5 (16.1%)		
2	95 (93.1%)	35 (100%)	36 (100%)	24 (77.4%)		
Bowels	1.96 ± 0.28	2.00 ± 0.00	2.00 ± 0.00	1.87 ± 0.50	4.63	
0	2 (2%)	0 (0%)	0 (0%)	2 (6.5%)		
1	0 (0%)	0 (0%)	0 (0%)	0 (0%)		
2	100 (98%)	35 (100%)	36 (100%)	29 (93.5%)		
Bladder	1.92 ± 0.36	2.00 ± 0.00	1.94 ± 0.33	1.81 ± 0.54	6.26*	1 > 3
0	3 (2.9%)	0 (0%)	1 (2.8%)	2 (6.5%)		
1	2 (2%)	0 (0%)	0 (0%)	2 (6.5%)		
2	97 (95.1%)	35 (100%)	35 (97.2%)	27 (87.1%)		
Mobility	2.91 ± 0.32	2.97 ± 0.17	3.00 ± 0.00	2.74 ± 0.51	13.49*	1 > 3, 2 > 3
0	0 (0%)	0 (0%)	0 (0%)	0 (0%)		
1	1 (1%)	0 (0%)	0 (0%)	1 (3.2%)		
2	7 (6.9%)	1 (2.9%)	0 (0%)	6 (19.4%)		
3	94 (92.2%)	34 (97.1%)	36 (100%)	24 (77.4%)		
Stairs	1.89 ± 0.31	1.94 ± 0.24	2.00 ± 0.00	1.71 ± 0.46	15.86**	1 > 3, 2 > 3
0	0 (0%)	0 (0%)	0 (0%)	0 (0%)		
1	11 (10.8%)	2 (5.7%)	0 (0%)	9 (29%)		
2	91 (89.2%)	33 (94.3%)	36 (100%)	22 (71%)		
Transfers	2.91 ± 0.38	3.00 ± 0.00	3.00 ± 0.00	2.71 ± 0.64	14.44*	1 > 3, 2 > 3
0	0 (0%)	0 (0%)	0 (0%)	0 (0%)		
1	3 (2.9%)	0 (0%)	0 (0%)	3 (9.7%)		
2	3 (2.9%)	0 (0%)	0 (0%)	3 (9.7%)		
3	96 (94.1%)	35 (100%)	36 (100%)	25 (80.6%)		
**Lawton IADL scale**	5.82 ± 2.41	7.54 ± 0.89	6.69 ± 1.28	2.87 ± 1.84	64.90**	1 > 3, 2 > 3
Shopping	0.56 ± 0.50	0.91 ± 0.28	0.69 ± 0.47	0.00 ± 0.00	59.30**	1 > 3, 2 > 3
0	45 (44.1%)	3 (8.6%)	11 (30.6%)	31 (100%)		
1	57 (55.9%)	32 (91.4%)	25 (69.4%)	0 (0%)		
Housekeeping	0.89 ± 0.31	1.00 ± 0.00	1.00 ± 0.00	0.65 ± 0.49	27.96**	1 > 3, 2 > 3
0	11 (10.8%)	0 (0%)	0 (0%)	11 (35.5%)		
1	91 (89.2%)	35 (100%)	36 (100%)	20 (64.5%)		
Finance-handling	0.84 ± 0.37	1.00 ± 0.00	1.00 ± 0.00	0.48 ± 0.51	43.04**	1 > 3, 2 > 3
0	16 (15.7%)	0 (0%)	0 (0%)	16 (51.6%)		
1	86 (84.3%)	35 (100%)	36 (100%)	15 (48.4%)		
Food preparation	0.54 ± 0.50	0.89 ± 0.32	0.56 ± 0.50	0.13 ± 0.34	37.57**	1 > 2 > 3
0	47 (46.1%)	4 (11.4%)	16 (44.4%)	27 (87.1%)		
1	55 (53.9%)	31 (88.6%)	20 (55.6%)	4 (12.9%)		
Transport mode	0.81 ± 0.39	0.94 ± 0.24	1.00 ± 0.00	0.45 ± 0.51	38.53**	1 > 3, 2 > 3
0	19 (18.6%)	2 (5.7%)	0 (0%)	17 (54.8%)		
1	83 (81.4%)	33 (94.3%)	36 (100%)	14 (45.2%)		
Telephone use	0.94 ± 0.24	1.00 ± 0.00	1.00 ± 0.00	0.81 ± 0.40	14.46**	1 > 3, 2 > 3
0	6 (5.9%)	0 (0%)	0 (0%)	6 (19.4%)		
1	96 (94.1%)	35 (100%)	36 (100%)	25 (80.6%)		
Laundry	0.63 ± 0.49	0.80 ± 0.41	0.78 ± 0.42	0.26 ± 0.45	25.78**	1 > 3, 2 > 3
0	38 (37.3%)	7 (20%)	8 (22.2%)	23 (74.2%)		
1	64 (62.7%)	28 (80%)	28 (77.8%)	8 (25.8%)		
Medication responsibility	0.61 ± 0.49	1.00 ± 0.00	0.67 ± 0.48	0.10 ± 0.30	56.51**	1 > 2 > 3
0	40 (39.2%)	0 (0%)	12 (33.3%)	28 (90.3%)		
1	62 (60.8%)	35 (100%)	24 (66.7%)	3 (9.7%)		

The listed statistics were mean ± standard deviation (SD) or frequency (percentage, %) as appropriate for the data type.

**Abbreviations:** NC = Normal control; MCI = Mild cognitive impairment; and Lawton IADL scale = Lawton Instrument Activities of Daily Living scale.

^a^ 1: Normal control group; 2: Mild cognitive impairment group; and 3: Dementia group.

^b^ The multiple comparisons among the three groups of subjects were conducted using the Dunn’s post hoc tests in the SPSS 19.0 software.

*p < 0.05.

**p < 0.001.

**Table 4:** Univariate analysis of the basic and instrumental activities of daily living functions among the subjects with mild, moderate, and severe dementia respectively.

Characteristics	Mild Dementia (*n* = 12)	Moderate Dementia (*n* = 13)	Severe Dementia (*n* = 6)	*χ*^*2*^ (*df* = 2)	Pairwise Comparison^a^^,^^b^
**Barthel Index (BI)**	19.58 ± 1.44	17.92 ± 2.18	13.67 ± 6.47	9.98**	1 > 3
Feeding	2.00 ± 0.00	1.92 ± 0.28	1.83 ± 0.41	1.84	
0	0 (0%)	0 (0%)	0 (0%)		
1	0 (0%)	1 (7.7%)	1 (16.7%)		
2	12 (100%)	12 (92.3%)	5 (83.3%)		
Grooming	1.00 ± 0.00	0.85 ± 0.38	0.33 ± 0.52	11.24**	1 > 3, 2 > 3
0	0 (0%)	2 (15.4%)	4 (66.7%)		
1	12 (100%)	11 (84.6%)	2 (33.3%)		
Toilet use	2.00 ± 0.00	1.77 ± 0.44	1.50 ± 0.84	4.09	
0	0 (0%)	0 (0%)	1 (16.7%)		
1	0 (0%)	3 (23.1%)	1 (16.7%)		
2	12 (100%)	10 (76.9%)	4 (66.7%)		
Bathing	0.92 ± 0.29	0.62 ± 0.51	0.17 ± 0.41	9.60**	1 > 3
0	1 (8.3%)	5 (38.5%)	5 (83.3%)		
1	11 (91.7%)	8 (61.5%)	1 (16.7%)		
Dressing	1.92 ± 0.29	1.77 ± 0.44	1.17 ± 0.98	4.74	
0	0 (0%)	0 (0%)	2 (33.3%)		
1	1 (8.3%)	3 (23.1%)	1 (16.7%)		
2	11 (91.7%)	10 (76.9%)	3 (50.0%)		
Bowels	2.00 ± 0.00	2.00 ± 0.00	1.33 ± 1.03	8.62*	1 > 3, 2 > 3
0	0 (0%)	0 (0%)	2 (33.3%)		
1	0 (0%)	0 (0%)	0 (0%)		
2	12 (100%)	13 (100%)	4 (66.7%)		
Bladder	1.92 ± 0.29	1.92 ± 0.28	1.33 ± 1.03	3.25	
0	0 (0%)	0 (0%)	2 (33.3%)		
1	1 (8.3%)	1 (7.7%)	0 (0%)		
2	11 (91.7%)	12 (92.3%)	4 (66.7%)		
Mobility	3.00 ± 0.00	2.69 ± 0.48	2.33 ± 0.82	6.61*	1 > 3
0	0 (0%)	0 (0%)	0 (0%)		
1	0 (0%)	0 (0%)	1 (16.7%)		
2	0 (0%)	4 (30.8%)	2 (33.3%)		
3	12 (100%)	9 (69.2%)	3 (50.0%)		
Stairs	1.92 ± 0.29	1.62 ± 0.51	1.50 ± 0.55	4.20	
0	0 (0%)	0 (0%)	0 (0%)		
1	1 (8.3%)	5 (38.5%)	3 (50%)		
2	11 (91.7%)	8 (61.5%)	3 (50%)		
Transfers	2.92 ± 0.29	2.77 ± 0.60	2.17 ± 0.98	4.98	
0	0 (0%)	0 (0%)	0 (0%)		
1	0 (0%)	1 (7.7%)	2 (33.3%)		
2	1 (8.3%)	1 (7.7%)	1 (16.7%)		
3	11 (91.7%)	11 (84.6%)	3 (50%)		
**Lawton IADL scale**	4.33 ± 1.44	2.31 ± 1.44	1.17 ± 1.17	13.69**	1 > 2, 1 > 3
Shopping	0.00 ± 0.00	0.00 ± 0.00	0.00 ± 0.00	0.00	
0	12 (100%)	13 (100%)	6 (100%)		
1	0 (0%)	0 (0%)	0 (0%)		
Housekeeping	0.92 ± 0.29	0.54 ± 0.52	0.33 ± 0.52	6.83*	1 > 3
0	1 (8.3%)	6 (46.2%)	4 (66.7%)		
1	11 (91.7%)	7 (53.8%)	2 (33.3%)		
Finance-handling	0.83 ± 0.39	0.31 ± 0.48	0.17 ± 0.41	9.58**	1 > 2, 1 > 3
0	2 (16.7%)	9 (69.2%)	5 (83.3%)		
1	10 (83.3%)	4 (30.8%)	1 (16.7%)		
Food preparation	0.33 ± 0.49	0.00 ± 0.00	0.00 ± 0.00	7.04*	1 > 2
0	8 (66.7%)	13 (100%)	6 (100%)		
1	4 (33.3%)	0 (0%)	0 (0%)		
Transport mode	0.75 ± 0.45	0.38 ± 0.51	0.00 ± 0.00	9.19**	1 > 3
0	3 (25.0%)	8 (61.5%)	6 (100%)		
1	9 (75.0%)	5 (38.5%)	0 (0%)		
Telephone use	1.00 ± 0.00	0.77 ± 0.44	0.50 ± 0.55	6.39*	1 > 3
0	0 (0%)	3 (23.1%)	3 (50.0%)		
1	12 (100%)	10 (76.9%)	3 (50.0%)		
Laundry	0.42 ± 0.52	0.15 ± 0.38	0.17 ± 0.41	2.49	
0	7 (58.3%)	11 (84.6%)	5 (83.3%)		
1	5 (41.7%)	2 (15.4%)	1 (16.7%)		
Medication responsibility	0.08 ± 0.29	0.15 ± 0.38	0.00 ± 0.00	1.12	
0	11 (91.7%)	11 (84.6%)	6 (100%)		
1	1 (8.3%)	2 (15.4%)	0 (0%)		

The listed statistics were mean ± standard deviation (SD) or frequency (percentage, %) as appropriate for the data type.

**Abbreviations:** Lawton IADL scale = Lawton Instrument Activities of Daily Living scale.

^a^ 1: Mild dementia group; 2: Moderate dementia group; and 3: Severe dementia group.

^b^ The multiple comparisons among the three groups of subjects were conducted using the Dunn’s post hoc tests in the SPSS 19.0 software.

*p < 0.05.

**p < 0.001.

Moreover, as shown in Table 4, we classified the 31 participants with dementia into three groups, mild (*n* = 12), moderate (*n* = 13), and severe dementia (*n* = 6), using the CDR scale global scores [4]. The BI scores were significantly higher in the mild dementia group than the severe dementia group, but the moderate dementia group was not significantly worse than the mild dementia group or significantly better than the severe dementia group. Nevertheless, the Lawton IADL scale scores were significantly higher in the mild dementia group than the moderate and severe dementia groups. Specifically, the grooming, bathing, bowels, and mobility subtest scores of BI were significantly higher in the mild dementia group than the moderate dementia group; whereas the grooming, and bowels subtest scores of BI were significantly higher in the moderate dementia group than the severe dementia group. And, the finance-handling, and food preparation subtest scores of the Lawton IADL scale were significantly higher in the mild dementia group than the moderate dementia group; whereas the housekeeping, finance-handling, transport mode, and telephone use subtest scores of the Lawton IADL scale were significantly higher in the mild dementia group than the severe dementia group.

## Discussion

This study demonstrated that the cut-off value of 81.2 years for age might be considered a threshold for the deterioration of daily living function in older adults in clinical practice. Moreover, cognitive instruments such as the CDR scale, Q*mci*, MoCA, and MMSE play complementary roles in predicting the changes in IADL functions for older adults. Remarkably, the Q*mci* is the most statistically significant factor in predicting the changes in IADL function for young older adults, as are the MoCA and MMSE for old older adults. In addition, we recruited participants not only with MCI but also with dementia to integrate the description of IADL and BADL functions in older adults with a spectrum of cognitive impairment. In the Lawton IADL scale, medication responsibility, shopping, and finance-handling are the optimal indicators for discriminating between the NCs and the participants with MCI, the participants with MCI and dementia, and the participants with mild and moderate dementia; however, in the BI, bathing and grooming are the optimal indicators for discriminating between the participants with MCI and dementia and the participants with moderate and severe dementia.

A multivariate analysis was conducted to assess the partial effects of all the relevant covariates, as presented in Table 1. As illustrated in Table 2, the higher the problem-solving score of the CDR and MoCA with age > 81.2 years and the lower the Q*mci* score with age ≤ 81.2 years and MMSE score with age > 81.2 years, the more likely it was that a deterioration in IADL function would occur. These findings could not only help us make predictions but also elucidate the complementary roles of the CDR, Q*mci*, MoCA, and MMSE. Notably, the Q*mci* plays a major role in predicting the changes in IADL function in “young” older adults, whereas the MoCA and MMSE play key roles in predicting the changes in IADL functions for “old” older adults.

Our results indicate that the capability to execute complex IADL may be a critical factor in the differentiation between NCs, patients with MCI, and patients with dementia. The Lawton IADL scale total scores in the NC group were not significantly higher than those in the MCI group (*p* = 0.07), but the total and all subtests scores of the Lawton IADL scale in the NC and MCI groups were significantly higher than those in the dementia group (*p* < 0.001). Furthermore, the best indicator of the Lawton IADL scale for differentiating between patients with MCI and the NCs was medication responsibility, followed by food preparation. The best indicator of the Lawton IADL scale for differentiating between patients with dementia and patients with MCI was shopping, followed by telephone use. These results are similar to those of a previous study demonstrating that the odd ratios of telephone use, medication taking, and finance-handling in the Lawton IADL scale are significantly different [23]. Therefore, the Lawton IADL scale may be preferable, particularly for older adults with a spectrum of cognitive impairment. According to these results, we suggest that participants lacking the ability to conduct IADL functions such as medication responsibility and shopping are at a greater risk of developing MCI and dementia, respectively.

This study also revealed that the maintenance of BADL is a critical factor for distinguishing between individuals with dementia and individuals with MCI. The BI scores were significantly higher in the MCI group than in the dementia group (*p* < 0.001). In addition, the best indicator of the BI to differentiate patients with dementia from patients with MCI was bathing, followed by climbing stairs. Based on these results, we suggest that the lack of ability to perform BADL functions such as bathing confers older adults with a greater risk of developing dementia.

Moreover, the Lawton IADL scale total scores in the mild dementia group were significantly higher than those in the moderate and severe dementia group. The best indicator of the Lawton IADL scale for discriminating between patients with mild dementia and patients with moderate dementia was finance-handling, followed by food preparation. Furthermore, the BI scores in the mild dementia group were significantly higher than those in the severe dementia group, but the moderate dementia group scores were not significantly lower than those of the mild dementia group or significantly higher than those of the severe dementia group. However, the best indicator of the BI to discriminate patients with moderate dementia and patients with severe dementia was grooming, followed by bowel control. In line with these results, we suggest that a lack of ability to execute daily functions such as finance-handling and grooming corresponds to a greater risk of developing moderate dementia and severe dementia, respectively.

The key contribution of this study is the idea that the cut-off value of 81.2 years for age might be considered a threshold for the deterioration of daily living functions in older adults in clinical practice. In addition, cognitive functions have been demonstrated to be helpful for predicting the changes in IADL function for clinical use. We recommend the use of the Q*mci* for IADL function change prediction in young older adults and the use of MoCA and MMSE for such predictions in old older adults. Furthermore, the BI and the Lawton IADL scale are useful assessment tools for activities of daily living to differentiate between NCs, patients with MCI, and patients with dementia. Notably, older adults who are unable to take medication responsibly, or to shop and bath may face a greater risk of developing MCI and dementia, respectively; in patients with dementia, a lack of ability to handle finances and groom may be representative of a greater risk of developing moderate dementia and severe dementia, respectively. In practice, for family members, observing older adults while they perform activities of daily living is necessary to perceive functional changes for the early detection and treatment of MCI or dementia.

This study has several limitations. First, the small sample size and number of participants with particular subtypes of MCI and dementia recruited may have limited the statistical power and generalizability of the findings. The statistically significant findings still deserve our attention, although inferences based on statistically nonsignificant findings should be conservative because of this lack of statistical power. Further studies should include a larger sample size and other types of MCI and dementia to avoid spectrum bias. Second, the potential response bias and proxy bias may influence the correctness of each response in the BI and Lawton IADL scale. This limitation is associated with the inherent challenges of an interview or survey study design, which does not allow for the administration of the actual performance of activities of daily living in real-life circumstances. Third, the recall bias may have an influence on the Lawton IADL scale at baseline. Not only the GAM analysis for detecting any nonlinear effects of continuous covariates (e.g., age) on the mean value of the change score in the Lawton IADL scale, but the regression analysis of the change score in the Lawton IADL scale (Table 2) was also affected by the measurements of the current and baseline Lawton IADL scale. Specifically, according to what the rater (Lee) knew about the backgrounds of the participants, the durations since the change of IADL functions began were approximately less than 6 months, 6 months−24 months, and 3 years−7 years for the NCs, the subjects with MCI, and the subjects with dementia respectively. Although recall bias is inevitable and the magnitude of the recall bias might depend on the current status of the subject (normal, MCI, and dementia), the changes in the eight-item Lawton IADL scale are relatively easier to recall as compared to the other scales measuring cognitive functions. In addition, the interpretations of the reasonable findings in the well-fitted linear regression model of the change score in the Lawton IADL scale reported in Table 2 (*R*^2^ = 0.8230) might reduce the concern in the measurement issue to some extent. Finally, the baseline and current Lawton IADL scale should ideally be measured at two separate time points in a prospective study, but the experience learned from this cross-sectional study might be considered as a feasible alternative in assessing the change score of the Lawton IADL scale for a screening purpose.

In conclusion, this study adds to the evidence that the description of IADL and BADL functions is integrated in older adults with a spectrum of cognitive impairment. Notably, the Q*mci* is the most statistically significant predictor of changes in IADL function for “young” older adults, as are the MoCA and MMSE for “old” older adults.
